# scFlowReport: A Reproducible Workflow for Comparative Downstream Biological Analysis of Single-Cell RNA-seq Data

**DOI:** 10.3390/biom16091288

**Published:** 2026-09-06

**Authors:** Nayoung Park, Hyewon Lee, Jaebum Kim

**Affiliations:** 1 Department of Biomedical Science and Engineering, Konkuk University, Seoul 05029, Republic of Korea; 2 Department of Cardiology, Medical Research Institute, Pusan National University Hospital, Busan 49241, Republic of Korea; 3 College of Medicine, Pusan National University, Yangsan 50612, Republic of Korea

**Keywords:** single-cell RNA sequencing (scRNA-seq), Seurat, workflow, pipeline, differential expression, cell–cell communication, gene regulatory network

## Abstract

Single-cell RNA sequencing (scRNA-seq) studies are frequently organized around comparisons—disease versus control, treatment response, or genetic perturbation—yet biological interpretation still depends on integrating multiple independent downstream analyses for differential expression, functional enrichment, regulatory network inference, and cell–cell communication analysis. Applying these tools consistently across comparisons typically requires substantial custom scripting, and their heterogeneous outputs must be manually harmonized before the results can be compared or reported together. We present scFlowReport, a lightweight, configuration-driven workflow that propagates a single user-defined comparison across cell-level and sample-aware pseudobulk differential expression, over-representation and ranked functional enrichment, transcription-factor regulon export for SCENIC, and group-resolved LIANA cell–cell communication analysis, starting from an already annotated Seurat object. The workflow automatically compiles complementary downstream results into standardized figures, summary tables, and a self-contained static HTML report that can be readily inspected and shared without requiring a persistent server. Application of scFlowReport to a publicly available Atopic Dermatitis scRNA-seq dataset demonstrated its utility by enabling researchers to obtain complementary biological evidence from multiple established downstream analyses. By coordinating complementary downstream analyses under a shared comparison framework, scFlowReport provides a practical and reproducible workflow for systematic interpretation of comparative single-cell transcriptomic data.

## 1. Introduction

Single-cell RNA sequencing (scRNA-seq) is now a standard method for resolving cellular heterogeneity and identifying cell-type-specific responses in development, disease, and treatment contexts [1,2]. Established frameworks such as Seurat [3,4] and Scanpy [5] support upstream processing steps, including quality control, normalization, integration, and clustering, making these upstream analyses broadly accessible. However, once cells are clustered and annotated, biological interpretation often requires multiple independent tools for differential expression, functional enrichment, regulatory network inference, and cell–cell communication analysis. These analyses are typically performed using separate packages without a unified downstream workflow [6,7]. Many scRNA-seq studies focus on explicit comparisons, such as disease versus control, treatment response, or other clinical contrasts [8] at single-cell resolution. Consistent downstream analysis across such comparisons is critical for interpretation. The lack of a standardized comparative workflow leads to repetitive scripting, inconsistent output formats, and reduced reproducibility.

Several integrated platforms and workflows have been developed to address these challenges, but they vary in scope and intended use. Web-based platforms such as ASAP [9], Granatum [10], and iS-CellR [11] offer graphical interfaces that guide users from raw data through preprocessing, clustering, and basic differential expression, primarily for those without programming expertise. More recent tools include SeuratExtend [12], which adds downstream features such as functional enrichment, trajectory inference, and gene regulatory network reconstruction to Seurat. Single Cell Portal [13] and CZ CELLxGENE Discover [14] focus on interactive exploration and public data sharing. In the R/Bioconductor ecosystem, iSEE [15] provides a Shiny interface for exploring SingleCellExperiment objects. These tools have improved accessibility and standardization. Each addresses different stages and use cases of single-cell analysis. A comparison of the scope and key characteristics of these frameworks is provided in Supplementary Table S1.

A related challenge concerns the downstream analytical modules themselves. Sample-aware pseudobulk analysis improves differential expression testing by controlling for pseudoreplication and increasing statistical power [16,17,18], while functional interpretation, transcriptional regulatory analysis, and cell–cell communication are typically performed using dedicated tools such as g:Profiler [19], GSEA [20], clusterProfiler [21], SCENIC [22], and LIANA [23] or CellChat [24]. Although these methods are individually mature and well validated, applying them consistently still requires substantial custom scripting. Moreover, downstream tools often produce heterogeneous outputs that differ in data structures, naming conventions, and statistical summaries [25,26,27], requiring additional manual harmonization before the results can be compared or reported together. Maintaining consistent comparison definitions across analytical modules presents a further challenge, as samples, cell types, and experimental conditions must be specified repeatedly throughout the workflow. Consequently, downstream analyses are frequently implemented as project-specific scripts, with parameters, software versions, intermediate outputs, and comparison definitions distributed across multiple scripts and notebooks. Such fragmentation reduces reproducibility and complicates result sharing. Automatically generating a portable, self-contained analysis report further improves transparency, facilitates result sharing, and supports reproducible dissemination.

To address these challenges, we developed scFlowReport, a lightweight, configuration-driven workflow for researchers who already hold a curated, cell-type-annotated Seurat object and need a single tool that applies consistent comparison definitions across differential expression, regulatory, and cell–cell communication analyses, harmonizes the results into standardized tables and figures, and emits a self-contained static HTML report that can be inspected and shared without a persistent web application. The primary contribution of scFlowReport lies in coordinating established downstream methods under a shared comparison definition, together with configuration-driven reproducibility and automated, standardized reporting, rather than in introducing new statistical methods. Accordingly, the present study focuses on workflow integration and reproducible comparative analysis rather than benchmarking the biological performance of individual downstream methods.

## 2. Materials and Methods

### 2.1. Workflow Overview

scFlowReport is a lightweight, configuration-driven workflow for comparative downstream analysis of previously processed single-cell RNA sequencing (scRNA-seq) data. This workflow assumes that upstream preprocessing, clustering, and cell-type annotation are completed in advance. The input Seurat object is expected to contain an RNA assay and completed cell-type annotations, together with metadata identifying biological samples and comparison groups; the corresponding metadata columns and comparison groups are specified through a YAML configuration file. A single user-defined comparison (group 1 versus group 2) is maintained consistently across all downstream modules.

Prior to downstream analysis, scFlowReport validates the configured assay and required metadata fields, confirms representation of both comparison groups, and performs compatibility preprocessing when necessary to ensure consistent handling of Seurat objects across versions. The workflow subsequently performs cell-type-resolved differential expression using both cell-level and sample-aware pseudobulk approaches, followed by cell–cell communication analysis with LIANA and transcription-factor regulon analysis with SCENIC. Functional enrichment analyses are performed on the principal outputs of the differential expression, communication, and regulon modules to facilitate downstream interpretation. Finally, the resulting tables, figures, and analysis metadata are compiled into a self-contained static HTML report. Any optional user-defined cell-type restriction is applied consistently across all applicable downstream modules.

### 2.2. Cell-Level Differential Expression Analysis

Cell-level differential expression analysis is conducted independently for each eligible cell type using Seurat::FindMarkers (v4.4.0) [3], with the configured group variable (group_col) defining the comparison groups. Eligibility for cell-level group comparisons requires a configurable minimum number of cells in each comparison group within the cell type (default: ≥20 cells per group). The min.pct parameter is supplied from the user configuration (default: min.pct = 0.1), and logfc.threshold is set to 0 to retain the complete test result before applying user-defined reporting filters. Differential expression results are organized into a common schema containing the comparison name, cell type, gene identifier, log2 fold change, raw and adjusted *p* values, group-specific detection fractions, and a direction label indicating higher expression in group 1 or group 2. Analyses of other cell types and downstream modules continue even when an individual comparison is ineligible or fails, with eligibility outcomes and skip reasons recorded explicitly.

Differential expression results are subsequently filtered according to the configured fold-change and adjusted *p*-value thresholds (default: |log2FC| ≥ 0.25 and adjusted *p* ≤ 0.05). Filtered differential expression results are summarized using cell-type-level DEG count bar plots, volcano plots, and heatmaps of selected genes. For heatmaps, the top n filtered DEGs in each direction are selected by adjusted *p* value, with absolute log2 fold change used to break ties (default: *n* = 10), and group-level values represent averages of per-sample standardized expression values. For functional enrichment analysis, filtered gene lists are analyzed by over-representation analysis using gprofiler2::gost (v0.2.4) [19], with the organism specified by the workflow configuration (default: hsapiens). Enrichment analysis is performed separately for genes upregulated in each comparison direction. Users can optionally restrict the background to genes detected in the corresponding cell type based on a configurable minimum detection fraction (default: 0.05), rather than using the complete annotated gene set [28]. Because g:Profiler is accessed through its online service, enrichment analysis uses the database release available through the g:Profiler API at the time of execution. The corresponding g:Profiler data and source database versions are recorded in the run metadata.

Separately, the complete differential expression results are used to construct gene-level rank vectors using a configurable ranking metric. The default metric is signed log2 fold change, with signed −log10 (adjusted *p* value) and a composite metric combining log2 fold change and −log10 (adjusted *p* value) also supported. The resulting vectors are sorted in decreasing order and analyzed using fgsea::fgsea (v1.36.2) [20] with a configured MSigDB collection (v26.1.0, default: Hallmark (H)) [29]. Alternatively, users can provide custom gene sets in Gene Matrix Transposed (GMT) format, allowing FGSEA to be performed using user-defined pathway collections or gene signatures. Enrichment results are visualized as dot plots for g:Profiler and normalized enrichment score bar plots for FGSEA, respectively.

### 2.3. Sample-Aware Pseudobulk Differential Expression Analysis

Sample-aware pseudobulk differential expression analysis is conducted independently for each eligible cell type to reduce pseudoreplication associated with cell-level testing. Raw counts are retrieved from the configured assay for each eligible cell type and aggregated across cells sharing the same biological sample identifier (sample_col) to produce a sample-level pseudobulk count matrix. Each sample-level pseudobulk profile is retained as an independent biological replicate in the subsequent edgeR model, with group membership defined by the corresponding sample metadata. Sample-by-cell-type units failing the minimum cell threshold are excluded before aggregation. Pseudobulk eligibility requires a minimum number of samples per comparison group and a minimum number of cells per sample-by-cell-type unit, as defined by the current run configuration (defaults: ≥3 samples per group and ≥10 cells per sample-by-cell-type unit). The resulting pseudobulk count matrix is analyzed using the edgeR (v4.8.2) [30] quasi-likelihood framework (DGEList, filterByExpr, calcNormFactors, estimateDisp, glmQLFit, and glmQLFTest). The contrast is defined such that positive log2 fold changes indicate higher expression in group 1, and negative values indicate higher expression in group 2. Differential expression results are organized into the same common schema as the cell-level analysis. The Benjamini–Hochberg-adjusted FDR reported by edgeR::topTags is used directly as the adjusted_*p*_value field, together with the comparison name, cell type, gene identifier, log2 fold change, raw *p* value, and direction label.

Differential expression results are filtered according to the configured fold-change and adjusted *p*-value thresholds (default: |log2FC| ≥ 0.25 and adjusted *p* ≤ 0.05). The resulting DEG tables are processed using the same downstream visualization and functional enrichment workflow as described for the cell-level analysis.

### 2.4. Transcription-Factor Regulon Analysis

Transcription-factor regulon analysis is conducted using pySCENIC through an export–execute–import workflow. For each selected cell type, genes are retained only if expressed in at least a user-configurable minimum fraction of cells (default: 1%), and the resulting cells-by-genes expression matrix is exported for pySCENIC analysis. pySCENIC (v0.12.1) [31] is executed independently for each cell type using the human hg38 RefSeq r80 cisTarget mc_v10_clust gene-based ranking database (TSS ± 10 kb) and the corresponding v10nr_clust motif-to-TF annotation resource to infer transcription-factor regulons and calculate per-cell regulon activity scores (AUCell). When both comparison groups satisfy the configured minimum cell requirement (default: ≥20 cells per group within the selected cell type), per-cell regulon activity scores are compared between groups for each regulon using Wilcoxon rank-sum tests, followed by Benjamini–Hochberg multiple-testing correction to identify differentially active regulons.

Differentially active regulons are filtered according to the configured ΔAUC and adjusted *p*-value thresholds (defaults: |ΔAUC| ≥ 0.03 and adjusted *p* ≤ 0.05). Target genes are derived from the adjacency tables using configurable importance-score filtering and top-target selection (defaults: top 200 targets per regulon after importance filtering at the 0.9 quantile), aggregated into direction-specific target sets within each cell type, and analyzed by over-representation analysis using gprofiler2::gost. The workflow additionally generates summary visualizations of regulon activity and differential activity analysis, together with g:Profiler enrichment dot plots for downstream reporting.

### 2.5. Cell–Cell Communication Analysis

Condition-resolved cell–cell communication analysis is conducted using LIANA (v0.1.14) [23] with its pre-generated Consensus ligand–receptor resource. The Seurat object is subset into group 1 and group 2, and communication inference is performed independently within each subset using the configured cell-type annotation as sender and receiver identities. If normalized expression data are not present in the assay, Seurat::NormalizeData is applied using log-normalization before conversion to SingleCellExperiment (v1.32.0) [32]; scater::logNormCounts (v1.38.0) [33] is subsequently applied when necessary to ensure that log-normalized expression values are available for downstream LIANA execution. Communication inference is performed with liana::liana_wrap, followed by liana::liana_aggregate. Raw and aggregated interaction tables are generated separately for each comparison group together with run-level metadata. The workflow additionally generates visualization-ready summaries, including condition-specific circos plots, interaction heatmaps, prioritized ligand–receptor pair summaries, and comparative interaction-count visualizations for downstream reporting.

Aggregated interaction results from the two groups are merged into a comparative interaction table in which prioritized interactions are classified as group1_specific, group2_specific, or shared_prioritized. For each interaction class, unique ligand genes and unique receptor genes are collected separately, and over-representation analysis is performed independently for each gene set using g:Profiler.

### 2.6. Application to a Representative Dataset

The dataset used for the representative application was derived from a previously constructed, integrated, and annotated single-cell RNA sequencing dataset of inflammatory skin diseases [34]. The integrated dataset was constructed from previously published psoriasis, hidradenitis suppurativa, and healthy control skin samples (Gene Expression Omnibus (GEO) accession numbers GSE151177 [35] and GSE220116 [36]) and atopic dermatitis and healthy control skin samples (GSE147424 [37]). For the scFlowReport demonstration, analyses were restricted to the atopic dermatitis and healthy control groups and to four annotated cell types (keratinocytes, *n* = 22,239; fibroblasts, *n* = 12,390; T cells, *n* = 5403; dendritic cells, *n* = 2784), comprising 42,816 cells from 28 samples. The workflow was executed using the default configuration.

In addition, workflow execution was verified using the built-in IFNB dataset [3] provided by Seurat as a workflow validation dataset.

### 2.7. Workflow Implementation

To enhance reproducibility and simplify software dependency management, scFlowReport uses a staged Docker-based execution model. R-based analyses and pySCENIC are executed in separate runtime environments using the r-analysis:v3 and pyscenic-custom:v1 Docker images, respectively. The primary R-based analysis environment uses R 4.5.3, with a separate Seurat v5 compatibility environment used for preprocessing when required, while the pySCENIC environment uses Python 3.10.20. This separation enables integration of tools with different software requirements into a single workflow while avoiding package compatibility conflicts.

Compatibility preprocessing is performed automatically as needed to ensure consistent handling of Seurat objects across supported versions. Run-specific configuration files and execution metadata are retained for each workflow execution, and intermediate files exchanged between stages are saved to the disk for inspection and reuse. After all analysis modules are complete, scFlowReport compiles the generated tables, figures, run metadata, and software provenance into a self-contained static HTML report and a portable report bundle for downstream inspection, sharing, and reproducible execution.

## 3. Results

### 3.1. Overview of the scFlowReport Workflow

**Figure 1:**
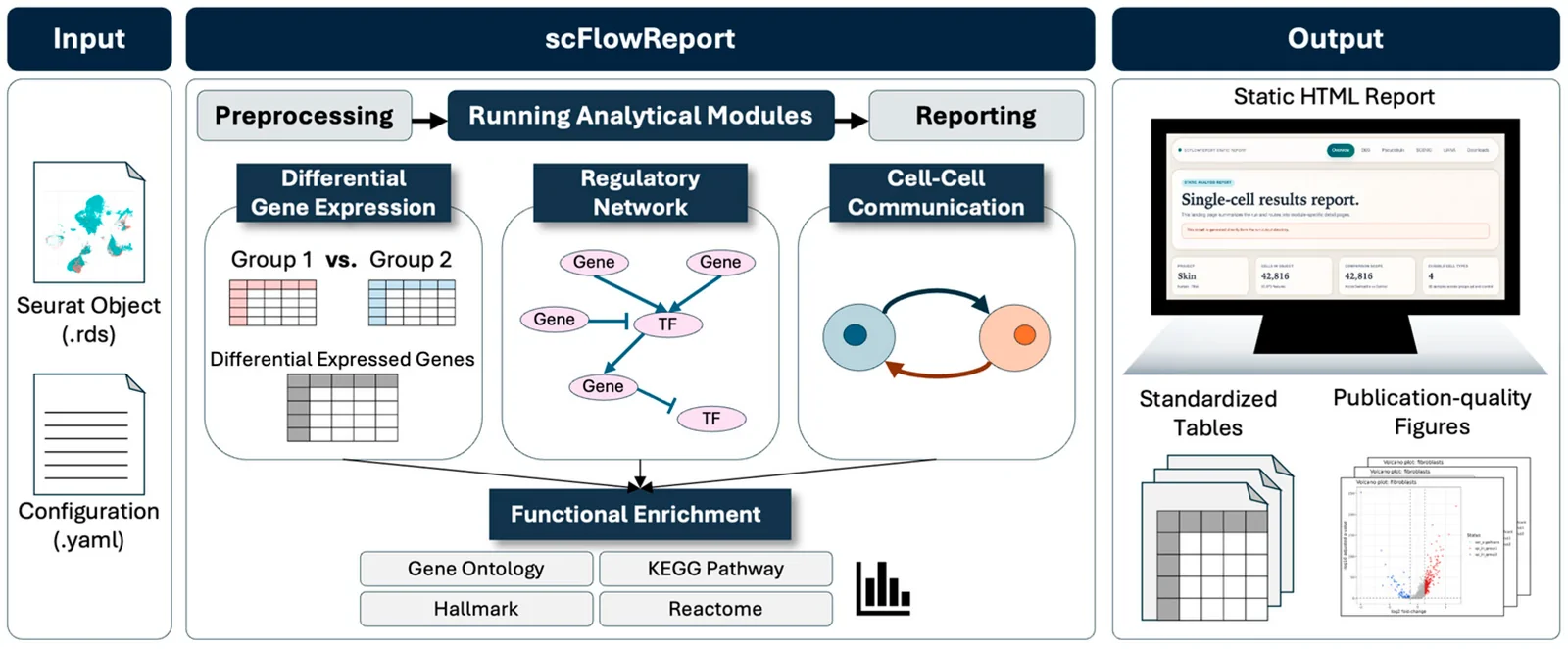
scFlowReport accepts a processed, cell-type-annotated Seurat object (.rds) together with a YAML configuration file as input and executes a three-stage workflow comprising preprocessing, analytical module execution, and report generation. The analytical stage includes cell-level and pseudobulk differential expression analysis, transcription factor regulon analysis, and cell–cell communication analysis. Functional enrichment analysis is subsequently performed. scFlowReport produces a standardized set of outputs, including a portable static HTML report, standardized result tables, and publication-quality figures.

The scFlowReport workflow generates a standardized collection of downstream analysis outputs from a single user-defined comparison, providing a consistent reporting structure across all supported analysis modules (Figure 1). A single workflow execution automatically performs comparison-level summarization, cell-level and sample-aware pseudobulk differential expression analyses, transcription-factor regulon analysis, and cell–cell communication analysis under a shared comparison definition. The resulting outputs are integrated into a unified reporting framework and, when enabled, automatically compiled into a self-contained static HTML report for downstream inspection and sharing.

**Figure 2:**
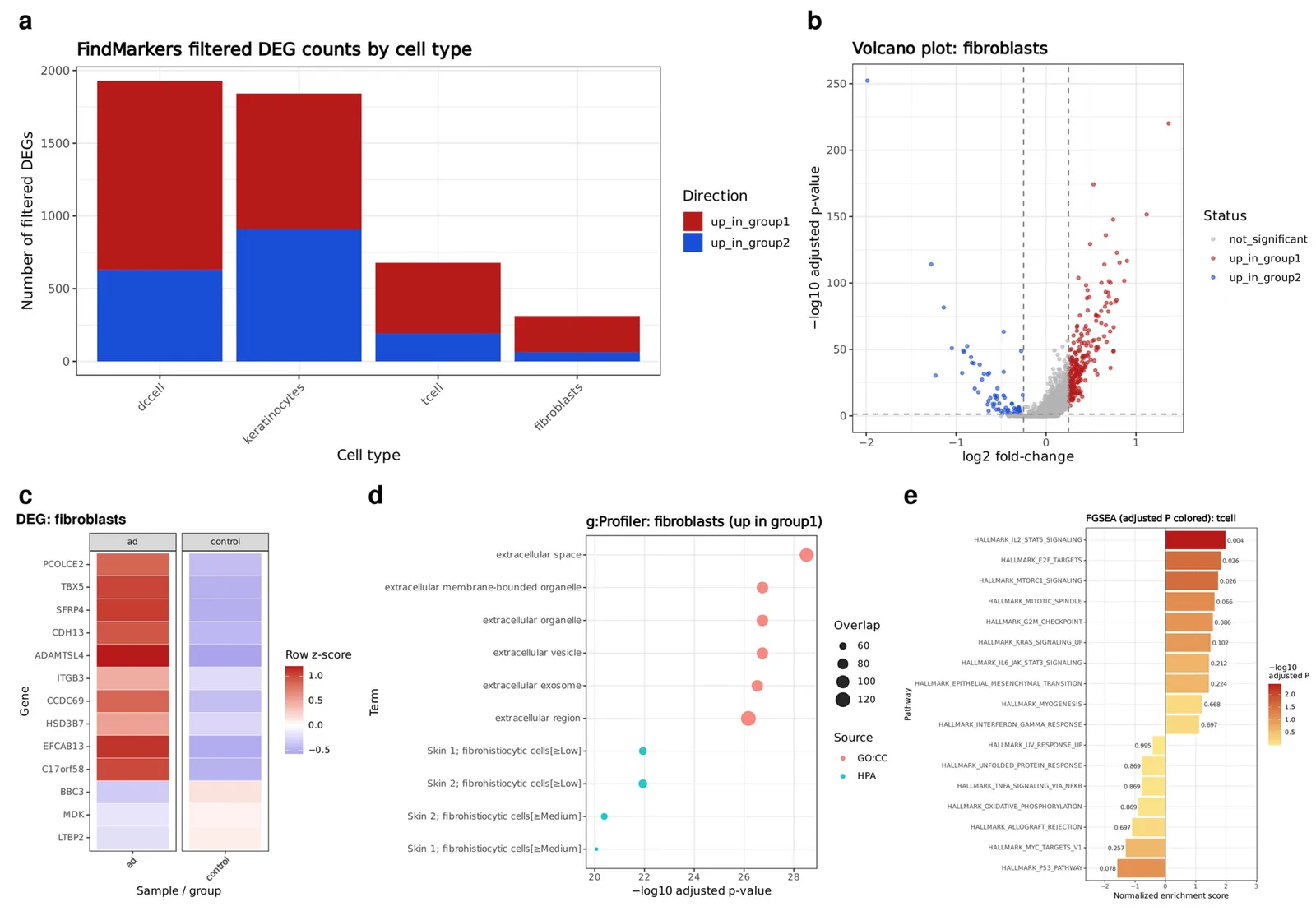
Representative differential expression analysis outputs. (**a**) Cell-type-level summary of filtered differentially expressed genes (DEGs), stratified by direction of change. (**b**) Example volcano plot showing differential expression results for a representative cell type. (**c**) Example heatmap of representative DEGs across the comparison groups. (**d**) Example over-representation analysis of genes up-regulated in one comparison group. (**e**) Example FGSEA results showing enriched Hallmark pathways in the two comparison directions.

For each cell type, the cell-level differential expression module generates complete and filtered DEG tables, together with standardized summary visualizations, including DEG count bar plots, volcano plots, heatmaps of selected markers, and DEG-linked enrichment results (Figure 2).

For eligible cell types, the pseudobulk module generates complete and filtered differential expression tables, along with standardized summary outputs, using a sample-aware analysis strategy. Output organization is consistent with the cell-level module (Figure 2).

**Figure 3:**
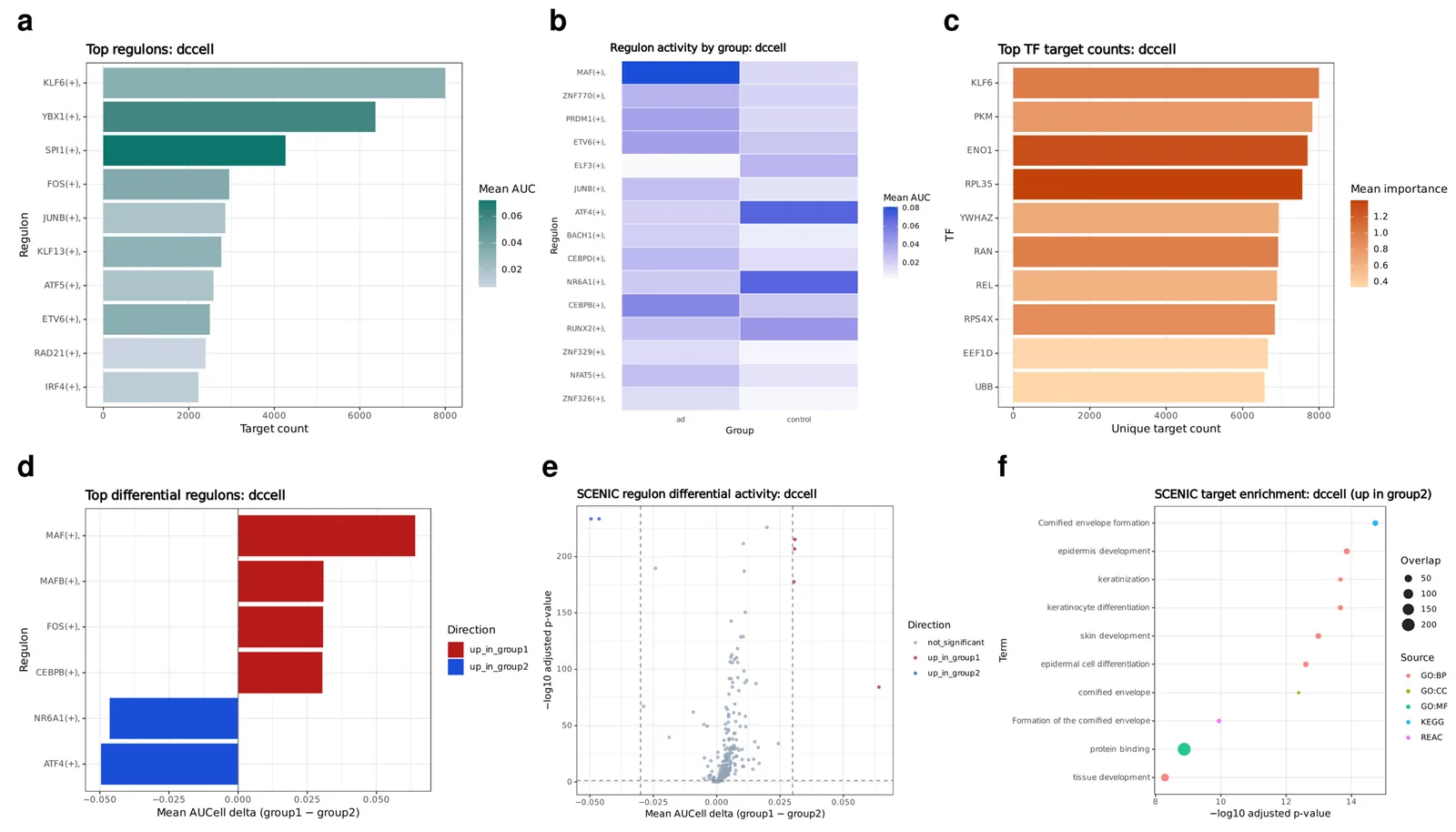
Representative SCENIC regulon analysis outputs. (**a**) Example summary of top regulons ranked by target gene count. (**b**) Example regulon activity heatmap across comparison groups. (**c**) Example summary of transcription factors ranked by target gene count. (**d**) Example differential regulon activity. (**e**) Example volcano plot of differential regulon activity. (**f**) Example functional enrichment of regulon target genes.

Following SCENIC analysis, the workflow produces per-cell-type regulon summary tables, adjacency-derived regulator summaries, and AUCell-based regulon activity matrices, together with summary visualizations including top-regulon bar plots, regulon activity heatmaps, and differential regulon and target-enrichment results when both comparison groups are present within a cell type (Figure 3).

**Figure 4:**
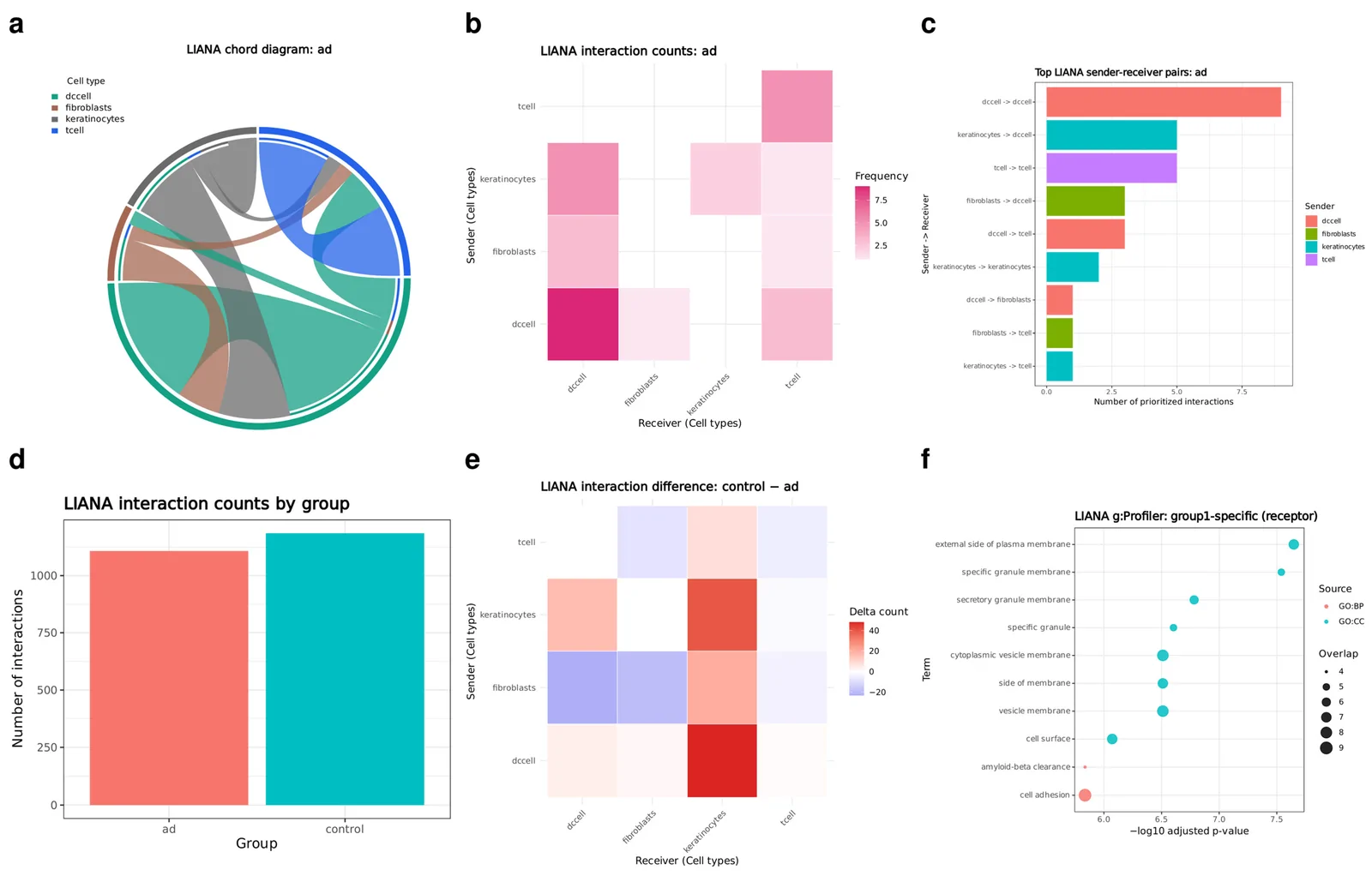
Representative LIANA cell–cell communication outputs. (**a**) Example chord diagram summarizing inferred cell–cell communication. (**b**) Example heatmap of interaction counts between sender and receiver cell types. (**c**) Example summary of prioritized sender–receiver cell-type pairs. (**d**) Comparison of the total number of inferred interactions between groups. (**e**) Example heatmap of between-group differences in interaction counts. (**f**) Example functional enrichment of receptor genes from group-specific interactions.

The LIANA module produces raw and aggregated interaction tables for each comparison group independently, together with condition-specific chord diagrams, interaction-count heatmaps, prioritized sender–receiver summaries, and comparative interaction-difference visualizations (Figure 4). A merged comparative table further classifies prioritized interactions as group1_specific, group2_specific, or shared_prioritized, accompanied by group-wise ligand and receptor enrichment results.

All of the above outputs are compiled into a static HTML report comprising a landing overview page and module-specific pages for DEG, pseudobulk, SCENIC, and LIANA, each linking directly to the underlying tables and figures, together with a portable, self-contained report bundle that preserves the same content outside of a persistent web application.

### 3.2. Workflow Evaluation

To assess practical workflow performance, scFlowReport was evaluated on two representative datasets differing in size, cell-type composition, and biological replication. The IFNB demonstration dataset contained 13,999 cells across 13 annotated cell types and one biological sample per comparison group, whereas the inflammatory skin dataset comprised 42,816 cells across four eligible cell types from 28 biological samples. These datasets were selected to illustrate workflow behavior under both limited- and sufficient-replication settings. Across both datasets, scFlowReport successfully generated standardized outputs for all applicable downstream modules according to the configured comparison definition.

Execution times varied substantially across workflow stages, with SCENIC and LIANA representing the largest computational components of the complete workflow (Table 1). The full IFNB workflow completed in approximately 62.4 min, whereas the skin dataset required approximately 5 h 56 min. Pseudobulk analysis was not performed for the IFNB dataset because it did not satisfy the default biological replication requirement. The substantially longer SCENIC runtime observed for the skin dataset likely reflects its larger per-cell-type expression matrices despite the smaller number of annotated cell types.

To further characterize computational resource requirements, peak memory usage was measured for each workflow execution stage using periodic docker stats sampling (Table 2). Across both datasets, pySCENIC exhibited the highest peak memory usage (9.0 GB for IFNB and 14.6 GB for skin), followed by r-analysis pass 1 (6.4 GB and 13.5 GB, respectively).

### 3.3. Application to a Public scRNA-seq Dataset

To demonstrate the complete downstream workflow on a biologically meaningful dataset, we applied the workflow to a previously processed, cell-type-annotated Seurat object derived from a published inflammatory skin dataset, restricted to the atopic dermatitis (AD) and healthy control groups and to four annotated cell types (keratinocytes, fibroblasts, T cells, and dendritic cells; 42,816 cells across 28 samples). Using the default configuration, scFlowReport completed cell-level and pseudobulk differential expression, DEG-linked enrichment, SCENIC regulon import and summarization, and group-wise LIANA communication analysis for all four eligible cell types and automatically assembled the resulting outputs into a single static HTML report.

**Figure 5:**
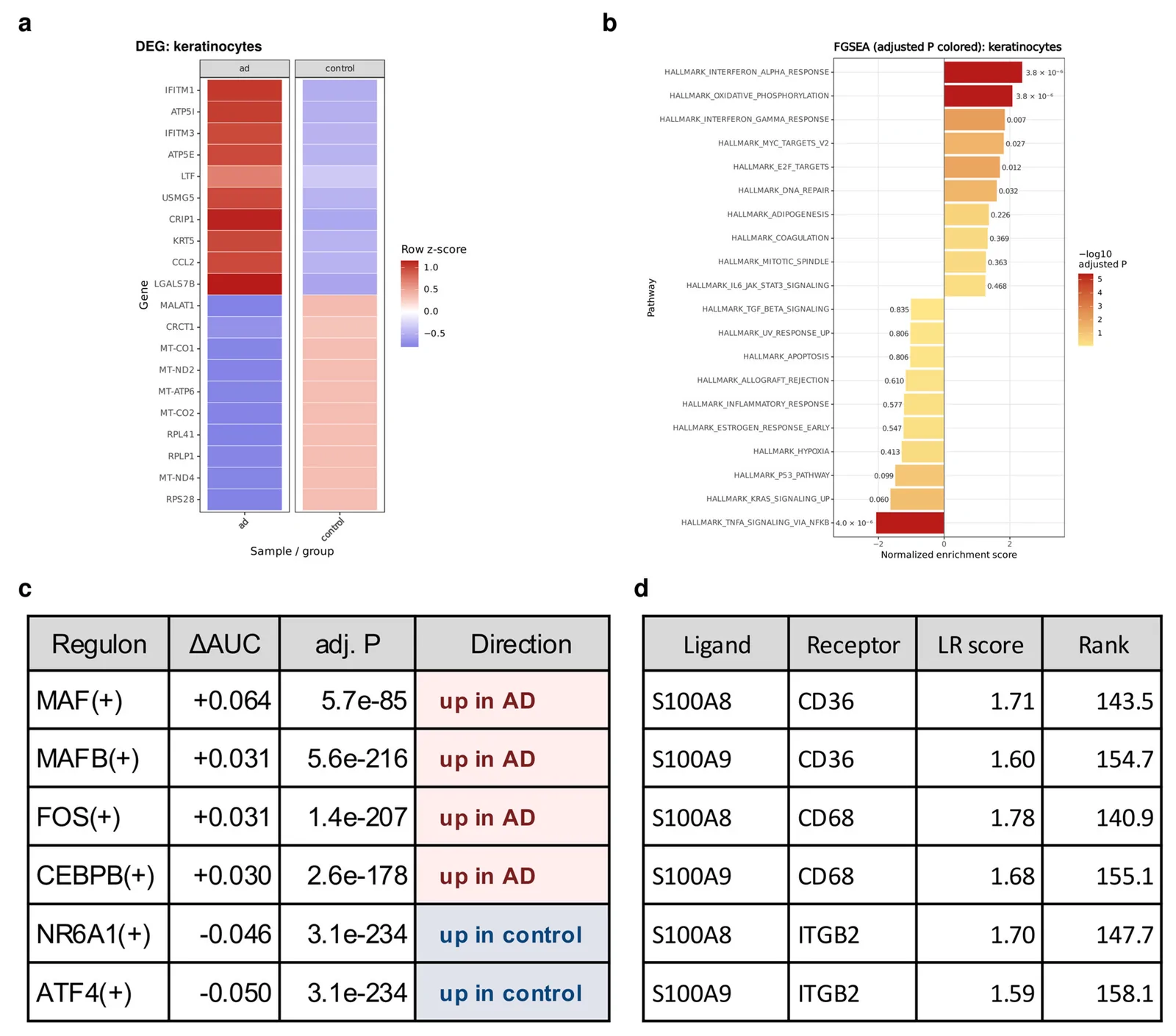
Representative outputs from application of scFlowReport to a public inflammatory skin dataset (AtopicDermatitis vs. Control). (**a**) Heatmap of representative keratinocyte differentially expressed genes. (**b**) Ranked gene set enrichment (FGSEA) of keratinocyte DEG results against Hallmark gene sets. Positive and negative normalized enrichment scores (NES) indicate enrichment in the AD and control directions, respectively, with bar color representing −log10(adjusted *p* value). (**c**) Differentially active SCENIC regulons in dendritic cells (dccell), from the filtered regulon differential activity table, showing mean AUCell activity difference (ΔAUC), Benjamini–Hochberg-adjusted *p* value, and direction of enrichment. (**d**) Selected AD-specific ligand–receptor pairs from the comparative LIANA interaction table for the keratinocytes → dccell axis, showing LIANA aggregate LR score and mean rank.

Across these complementary layers, scFlowReport recovered signals consistent with known epithelial and inflammatory biology in AD skin. Cell-level differential expression in keratinocytes identified AD-associated genes including KRT16, IFITM1, IFITM3, CCL2, and CCL27, alongside control-associated genes such as SBSN, LCE3D, and KRT17 (Figure 5a, Supplementary Table S2). FGSEA in the same cell type identified significant AD-associated enrichment of interferon-alpha and interferon-gamma response Hallmark gene sets, whereas TNFα signaling via NF-κB was significantly enriched in the control-associated direction (Figure 5b, Supplementary Table S3). The AD-associated interferon response is consistent with previous transcriptomic studies reporting type 1/interferon-related inflammatory programs in AD skin and keratinocytes [38,39]. SCENIC regulon analysis identified cell-type-specific transcription factor activity, including increased MAFB(+), FOS(+), and CEBPB(+) regulon activity in dendritic cells in the AD group (Figure 5c, Supplementary Table S4). Although this specific regulon combination has not, to our knowledge, been established as an AD-specific signature, MAFB has been implicated in the transcriptional regulation of macrophage-versus-dendritic-cell fate [40], whereas CEBPB has been identified as a regulator of monocyte-derived dendritic-cell chromatin states [41]. LIANA analysis identified AD-specific keratinocyte-to-dendritic-cell interactions, with both S100A8 and S100A9 prioritized as ligands toward CD36, CD68, and ITGB2 across a narrow rank range, indicating a coordinated alarmin-signaling axis rather than an isolated pair (Figure 5d, Supplementary Table S5). Taken together, these outputs demonstrate how a single scFlowReport run brings together cell-type-specific differential expression, pathway-level interpretation, regulon activity, and predicted intercellular signaling within a standardized reporting framework. These independently derived outputs can be considered together to support biological interpretation and hypothesis generation.

## 4. Discussion

The interpretation of comparative single-cell transcriptomic datasets increasingly depends on integrating complementary downstream analyses. These analyses are frequently performed using independent tools in heterogeneous execution environments. Because analytical modules capture complementary aspects of cell-type-specific biology, coordinating them within a consistent analytical framework provides a more systematic understanding of molecular responses than relying on individual analyses. This study introduces scFlowReport, a comparison-centered workflow that coordinates established downstream analyses within a unified execution framework. Maintaining a shared comparison definition reduces analytical inconsistencies that may occur when downstream analyses are configured independently across multiple tools. The workflow prioritizes consistent comparison management, reproducible execution, and integrated reporting, enabling the generation of complementary analyses from a processed and annotated Seurat object with minimal manual intervention.

In practical research contexts, these design principles reduce the technical complexity associated with comparative downstream analyses. Instead of repeatedly executing multiple independent tools, maintaining separate comparison definitions, and organizing outputs across different software environments, investigators can perform complementary downstream analyses within a single configuration-driven workflow while maintaining transparent execution records. This approach distinguishes scFlowReport from existing upstream-oriented platforms and interactive exploration tools, which primarily support data processing or visualization rather than coordinated downstream execution. The added value of scFlowReport therefore lies in reducing the manual coordination required to apply established downstream methods consistently and to organize their outputs within a reproducible reporting framework. An application to a publicly available inflammatory skin dataset restricted to AD and healthy control samples demonstrated the practical utility of the workflow by generating biologically interpretable downstream results from a processed Seurat object in a real comparative analysis context. In this application, the individual analytical modules provided complementary views of the same disease comparison: differential expression characterized cell-type-specific transcriptional changes, FGSEA placed these changes in a pathway-level context, SCENIC identified differential regulatory activity, and LIANA highlighted candidate intercellular signaling. For example, AD-associated interferon responses in keratinocytes, altered regulon activity in dendritic cells, and predicted keratinocyte-to-dendritic-cell signaling involving S100A8/S100A9 collectively highlight transcriptional, regulatory, and intercellular aspects of epithelial–immune alterations in AD skin. These independently derived results do not constitute a causal model, but their coordinated presentation facilitates hypothesis generation across transcriptional, regulatory, and intercellular levels.

Although the current implementation provides a reproducible core workflow, several limitations remain. The workflow is currently configured for human-oriented reference resources, and extending support to additional model organisms will require the integration and validation of corresponding organism-specific resources across downstream modules. Each execution presently supports a single predefined comparison; enabling multiple named comparisons represents an important future extension. Additionally, the current implementation is limited to transcriptome-based scRNA-seq analysis and does not yet support additional single-cell modalities, such as scATAC-seq or multimodal datasets.

Future development will prioritize expanding support for multiple named comparisons, improving organism compatibility, and incorporating additional single-cell modalities. Further objectives include closer integration of external regulatory analysis modules and broader benchmarking across diverse biological datasets. These enhancements will increase the range of biological questions and study designs that can be addressed within a unified downstream workflow while maintaining an emphasis on reproducible, comparison-centered analysis.

## 5. Conclusions

We presented scFlowReport, a lightweight, configuration-driven workflow for comparative downstream analysis of annotated single-cell RNA-seq data. The workflow applies a single comparison definition across cell-level and sample-aware pseudobulk differential expression, functional enrichment, transcription-factor regulon analysis, and group-resolved cell–cell communication analysis. It coordinates these complementary modules within a unified workflow and organizes the resulting outputs into a self-contained static HTML report.

The workflow provides a unified execution framework that applies consistent comparison definitions across complementary downstream analyses, transparently handles module-specific eligibility criteria, and records unsupported analyses without interrupting the overall workflow. These capabilities facilitate practical, reproducible, and comparison-centered downstream analyses that support biological interpretation of single-cell transcriptomic data.

## Figures and Tables

**Table 1:** Runtime of major scFlowReport workflow modules for the IFNB and skin demonstration datasets.

Workflow Stage	IFNB	Skin
Preprocessing	1.3 s	2 m 55.1 s
Cell-level DEG	14 m 34.5 s	58 m 50.0 s
Pseudobulk DEG	-	2 m 13.5 s
SCENIC	19 m 1.4 s	275 m 31.7 s
LIANA	27 m 27.6 s	15 m 27.0 s
Report Generation	39.1 s	~18.5 s
Workflow overhead *	36.4 s	1 m 7.6 s
Total workflow time	62 m 21.0 s	~5 h 56 m

* Workflow overhead includes orchestration, intermediate file handling, and transitions between analysis modules.

**Table 2:** Peak memory usage of major scFlowReport execution stages for the IFNB and skin demonstration datasets.

Execution Stage	IFNB	Skin
Seurat preparation	–	2.1 GB
r-analysis pass 1 *	6.4 GB	13.5 GB
pySCENIC	9.0 GB	14.6 GB
r-analysis pass 2 **	3.0 GB	4.5 GB

* r-analysis pass 1 includes overview generation, differential expression, functional enrichment, LIANA, and SCENIC input generation. ** r-analysis pass 2 includes SCENIC result processing and final report generation.

## Data Availability

All datasets analyzed in this study are publicly available from the Gene Expression Omnibus (GEO), as described in Methods. The scFlowReport source code, execution instructions, and complete software environment information are available at https://github.com/ibclab-hub/scFlowReport (accessed on 2 September 2026). An example YAML configuration file with default parameter settings (config.example.yaml) is provided in the repository. An example report bundle generated from the representative analysis, including the static HTML report and its associated result files, is also available at https://ibclab-hub.github.io/scFlowReport/example/Skin/ (accessed on 2 September 2026). The Docker images used for R-based analyses and pySCENIC are publicly available at https://hub.docker.com/layers/nayoungpark/r-analysis/v3/ (accessed on 2 September 2026) and https://hub.docker.com/layers/nayoungpark/pyscenic-custom/v1/ (accessed on 2 September 2026), respectively.

## Supplementary Materials

The following supporting information can be downloaded at: https://www.mdpi.com/article/10.3390/biom16091288/s1, Supplementary Table S1: Comparison of scFlowReport with representative single-cell analysis frameworks; Supplementary Table S2: Complete cell-level differential expression results for the Atopic Dermatitis vs. Control comparison; Supplementary Table S3: Complete FGSEA results for the Atopic Dermatitis vs. Control comparison; Supplementary Table S4: Differentially active SCENIC regulons in dendritic cells; Supplementary Table S5: Comparative LIANA ligand–receptor interaction results.

## Abbreviations

The following abbreviations are used in this manuscript:

scRNA-seq	Single-cell RNA sequencing
DEG(s)	Differentially expressed gene(s)
FDR	False discovery rate
AD	Atopic dermatitis
YAML	YAML Ain’t Markup Language
HTML	HyperText Markup Language
MSigDB	Molecular Signatures Database
AUC	Area under the (recovery) curve—regulon activity score computed by AUCell
LR	Ligand–receptor
IFNB	Interferon-beta-stimulated PBMC dataset (built-in Seurat/SeuratData example dataset)
